# Detecting Intestinal Goblet Cells of the Broadgilled Hagfish *Eptatretus cirrhatus* (Forster, 1801): A Confocal Microscopy Evaluation

**DOI:** 10.3390/biology11091366

**Published:** 2022-09-17

**Authors:** Alessio Alesci, Simona Pergolizzi, Serena Savoca, Angelo Fumia, Angelica Mangano, Marco Albano, Emmanuele Messina, Marialuisa Aragona, Patrizia Lo Cascio, Gioele Capillo, Eugenia Rita Lauriano

**Affiliations:** 1 Department of Chemical, Biological, Pharmaceutical and Environmental Sciences, University of Messina, 98166 Messina, Italy; 2 Department of Biomedical, Dental and Morphological and Functional Imaging, University of Messina, 98125 Messina, Italy; 3 Institute of Marine Biological Resources and Biotechnology, National Research Council (IRBIM, CNR), 98164 Messina, Italy; 4 Department of Clinical and Experimental Medicine, University of Messina, Padiglione C, A. O. U. Policlinico “G. Martino”, 98124 Messina, Italy; 5 Department of Veterinary Sciences, University of Messina, 98168 Messina, Italy

**Keywords:** confocal microscopy, *Eptatretus cirrhatus*, goblet cells, gut, immune system

## Abstract

**Simple Summary:**

The intestinal epithelium of fish, similar to mammals, consists mainly of enterocytes and goblet cells. Goblet cells play a key role in the secretion of mucus, which, in addition to promoting the digestion of nutrients, is the first protective barrier against bacteria, viruses, and pathogens. Our study aims to evaluate the presence, localization, and co-localization of 5-HT, TLR2, iNOS, and Piscidin1 in goblet cells of the intestine of *Eptatretus cirrhatus*. The results obtained by confocal microscopy show, for the first time, the positivity of goblet cells to the antibodies tested, suggesting the involvement of these cells in the intestinal immunity of broadgilled hagfish.

**Abstract:**

The fish intestine operates as a complicated interface between the organism and the environment, providing biological and mechanical protections as a result of a viscous layer of mucus released by goblet cells, which serves as a barrier against bacteria, viruses, and other pathogens, and contributes to the functions of the immune system. Therefore, goblet cells have a role in preserving the health of the body by secreting mucus and acting as sentinels. The ancient jawless fish broadgilled hagfish (*Eptatretus cirrhatus*, Forster, 1801) has a very basic digestive system because it lacks a stomach. By examining the presence, localization, and co-localization of 5-HT, TLR2, iNOS, and Piscidin1, this study intends to provide insight into the potential immune system contributions arranged by the gut goblet cells of broadgilled hagfish. Our results characterize intestinal goblet cells of broadgilled hagfish, for the first time, with the former antibodies, suggesting the hypothesis of conservation of the roles played by these cells also in primitive vertebrates. Moreover, this study deepens the knowledge about the still little-known immune system of hagfish.

## 1. Introduction

The fish intestine is one of the organs that is most frequently in the focus of scientific research, especially given its considerable complexity and connections with other organs and overall health in general [1]. This represents a complex interface between the organism and its environment; regulates the uptake of water, electrolytes, and nutrients from the lumen; and contributes to the neuroendocrine and immunological systems by defending the organ against pathogens and xenobiotics [2,3]. Histologically, it shares many characteristics with its mammalian counterpart [2,4]. The intestinal mucosa generally presents a mono-layered epithelium composed mainly of enterocytes, goblet cells, and neuroendocrine cells, which rests on a simple lamina propria, with nerves, blood vessels, extracellular matrix, and immune cells, such as macrophages, mast cells, dendritic cells, lymphocytes, and plasma cells [2,5,6,7,8], forming a gut-associated lymphoid tissue (GALT). Furthermore, goblet cells secrete a viscous layer of mucus that covers the intestinal surface, working to protect the organ both mechanically and biologically [4]. The intestinal epithelium thus represents a barrier against bacteria, viruses, and other pathogens [9]. Mucus consists mainly of glycoproteins and its excretion may be regulated by several stimuli, such as Toll-like receptor (TLR) signals [10,11]. TLRs are transmembrane proteins, belonging to pattern recognition receptors (PRRs), located on the surface of cells and in endosomes, which recognize bacteria, viruses, pathogens, and damage-associated molecular patterns (DAMPs). Several immune molecules may be present in the mucus, such as antimicrobial peptides (AMPs), cytokines, and chemokines, whose secretion may also depend on TLR-dependent stimuli [10,11,12]. In fish, the mucus secreted by goblet cells is rich in AMPs, such as piscidins, which are antibacterial molecules that are able to act indifferently against Gram-positive and Gram-negative bacteria [13]. Piscidins play a crucial role in the fish defense system, being present in different types of immune cells, such as mast cells and rodlet cells [2,14]. Goblet cells, therefore, play a role in maintaining the health of the body, through the secretion of mucus, and functioning as sentinels. Recently, a study by Zhang et al. (2020) suggested that some goblet cells, called sentinel-goblet cells (senGCs), by activating TLR signals, contribute to the immune response, also exacerbating mucus production [10,15]. Moreover, interestingly, goblet cells are involved in antigenic presentation to dendritic cells in the lamina propria of the intestine. Through stimulation by neurotransmitters such as acetylcholine, serotonin, and nitric oxide, goblet cells regulate their mucus secretion; furthermore, they can form goblet-cell-associated antigen passages (GAPs), allowing for the exposure of luminal antigens to antigen-presenting cells (APCs) in the lamina propria [9,15,16]. Serotonin (5-hydroxytryptamine; 5-HT) and nitric oxide (NO) regulate the secretion of goblet cells both under physiological and pathological conditions, and, apart from stimulating intestinal contractility and motility, can activate receptors on goblet cells to excite mucus production [17,18]. NO synthetase (NOS) is present in three different forms: n-neuronal, e-endothelial, and i-inducible. iNOS is present in almost all immune cell types, mainly phagocytes, but also in enterocytes and goblet cells. Losada et al. (2012), in a study on *Psetta maxima*, revealed goblet cells immunopositive to iNOS, suggesting that these cells are involved in the secretion of this chemical mediator [19,20].

The broadgilled hagfish (*Eptatretus cirrhatus*, Forster, 1801) is one of the ancient jawless fish—it possesses a very simple digestive system, lacking a proper stomach [21]. Hagfish presents an intestinal tract divided into three layers: mucosa, submucosa, and muscolaris serosa. The mucosa is rich in enterocytes; zymogenous cells, which facilitate digestion; and large goblet cells, as reported in our previous study [22].

This study aims to characterize the intestinal goblet cells of broadgilled hagfish by confocal microscopy, using the following antibodies, 5-HT, TLR2, iNOS, and Piscidin1, for the first time.

## 2. Materials and Methods

### 2.1. Samples

Samples of gut from three broadgilled hagfish were taken from our laboratory’s histotheca and were processed according to standard procedures to create long preparations for optical microscopy and paraffin block storage [2,22,23].

### 2.2. Tissue Preparation

The samples were fixed in 4% paraformaldehyde in 0.1 M phosphate-buffered saline (pH 7.4), rinsed in xylene, and embedded in Paraplast^®^ (McCormick Scientific LLC, St. Louis, MO, USA) after being dehydrated in graded ethanol for 12 to 18 h. Finally, using a rotary microtome (LEICA 2065 Supercut, Nussloch, Germany), serial slices (3–5 μm thick) were cut.

### 2.3. Histology and Histochemistry

Serial slices were stained for light microscopic examination using the Alcian Blue pH 2.5-PAS (04-163802 Bio-Optica Milano S.p.A., Milan, Italy) and Mallory Trichrome (04-020802 Bio-Optica Milano S.p.A., Milan, Italy) techniques [23,24].

### 2.4. Immunofluorescence

Serial slices were first deparaffinized gradually and then rehydrated in PBS before being blocked with 2.5% bovine serum albumin (BSA) for an hour. The sections were tested singularly and in double-label experiments after being subjected to primary antibodies against 5-HT, TLR2, iNOS, and Piscidin1 in a humid chamber overnight at 4 °C. [24]. Alexa Fluor 594 donkey anti-rabbit IgG TRITC conjugated and Alexa Fluor 488 donkey anti-mouse IgG FITC conjugated secondary antisera (Molecular Probes, Invitrogen, Eugene, OR, USA, 1:300) were used. The sections were mounted with Vectashield (Vector Labs, Burlingame, CA, USA) to avoid photobleaching, and the cover was slipped after washing [21]. The tissue preparations used as negative controls were made inactive by removing the primary antibodies. As a positive control, rat intestinal samples were employed to confirm the immunopositivity of the primary antibodies. Table 1 summarizes the information on antibodies.

### 2.5. Laser Confocal Immunofluorescence

With the help of a Zeiss LSM DUO confocal lasers scanning microscope with a META module, sections were analyzed, and pictures were taken (Carl Zeiss MicroImaging GmbH, Jena, Germany). Two helium–neon lasers (543 and 633 l) and two argon lasers (458 and 488 l) are included in this microscope [25]. A 2048 by 2048 pixel array with an 8-bit resolution was created by digitizing each image. Using helium–neon (543 nm) and argon (458 nm) lasers with a scanning speed of 1 min and 2 s and up to eight averages, optical slices of fluorescence samples were obtained. The images were refined using Zen 2011 (LSM 700 Zeiss software, Oberkochen, Germany). Each picture was quickly taken to prevent photo degradation. Digital photo cropping was conducted in Adobe Photoshop CC (Adobe Systems, San Jose, CA, USA) to build the figure montage [21]. The intensity profile of an image was displayed on a freely selected line using the “display profile” feature of the laser scanning microscope. The intensity curves are presented alongside the scanned images in graphs.

### 2.6. Quantitative Analysis

To collect information for the quantitative analysis, 5 sections and 10 fields were examined for each sample. Based on how positively the cells responded, observation fields were selected. Each field was assessed utilizing ImageJ software [26]. The image was converted to 8 bits, the background was cleared, and the cells were recognized using a “Threshold” filter and a mask. The cells were then counted using the “Analyze particles” plug-in. The number of goblet cells that were positive for 5-HT, TLR2, iNOS, and Piscidin1 in each field was quantified using SigmaPlot version 14.0 (Systat Software, San Jose, CA, USA). One-way ANOVA followed by Student’s t-test were used to analyze the normally distributed data. Data are shown as mean values and standard deviations (SD). Statistical significance was given to the following *p* values in this order: ** *p* ≤ 0.01, * *p* ≤ 0.05.

### 2.7. Phylogenetic Conservation of Primary Antibodies

Studies comparing several species have demonstrated how well-conserved 5-HT functions are in vertebrates [27]. From Agnatha to humans, the palmitoylation sites on the 5-HT receptor have been substantially conserved. All GPCR-type 5-HT receptor isoforms have been developed to preserve seven transmembrane segments with the N- and C-terminal extracellular and cytoplasmic domains. The C-terminal «-IIKCKFCRQ-stop» sequence of the orthologous sea lamprey 5-HT1A receptor is 100% similar to that of the human receptor. The 5-HT1A receptor’s C-terminal palmitoylation sites are completely preserved across the vertebrate lineage, from the Agnatha superclass (jawless fish) to the Gnathostomata superclass (jawed vertebrates) [28]. The Fugu 5-HT type 1 receptor genes were cloned and sequenced by Yamaguchi et al. using polymerase chain reaction (PCR) with degenerate primers, followed by phage library screening. The deduced amino acid sequences showed that the human 5-HT1A receptor shared the most similarities with F1A and F1A (71.5% and 63.7%, respectively). F1D, a different clone, had the most homology to the human type 1D receptor (70.5%) [29]. The 5-HT2C receptor gene of zebrafish and the 5-HT2C receptor of mammals have similarities that have been discovered [30].

TLRs are present in all vertebrate classes [31,32] and are highly conserved receptors from a physical and genetic perspective [2], playing a role in immunological response [33,34]. TLRs, particularly TLR2, have been detected in urochordates (*Styela plicata)* [35], cartilaginous fish [22], bony fish [14], and other upper vertebrates [32]. One part of the innate immune system that has been phylogenetically well-conserved is TLRs. In a study by Anandhakumar (2012), a 270 bp amplicon was amplified using a degenerate primer strategy that corresponded to the Toll/IL-1 (TIR) domain of TLR2 (GenBank ID: JF792 813). According to BLAST analysis, the maximum nucleotide identity with the TLR2 of mammals and upper teleost was 87% and 76%, respectively [36]. By comparing the TLR profiles of two fish species (*Danio rerio* and *Takifugu rubripes*), a collection of orthologous genes with substantial sequence conservation in human TLRs were identified [37,38].

An ancestral molecule called nitric oxide synthetase is crucial for the survival and growth of many biological systems, including the immunological, neurological, and cardiovascular systems. In vertebrates, three paralogous NOS genes have been identified: NOS1 (or nNOS) [39], NOS2 (or iNOS) [40], and NOS3 (or eNOS) [41,42]. In a recent study, Annona et al. discovered the expression of this molecule in many cyclostome tissues, including the brain, dorsal midline epidermis, tailbud, mouth, and cloaca (jawless vertebrates, including lamprey and hagfish). The three NOS isoforms are preserved among Agnatha and Gnathostomata, according to phylogenetic research. [43,44]. A study by Saeij et al. (2000) showed that carp iNOS encodes an 1127-aminoacid protein with 57% sequence identity to human iNOS [45].

In this investigation on broadgilled hagfish, Piscidin1 was utilized after being directly created in fish.

Therefore, a variety of vertebrate classes, from fish to mammals, have a high level of conservation for the investigated antibodies (5-HT, TLR2, and iNOS). The phylogenetic maintenance of these antibodies is supported by the fact that their receptors also match in mammals.

## 3. Results

**Figure 1:**
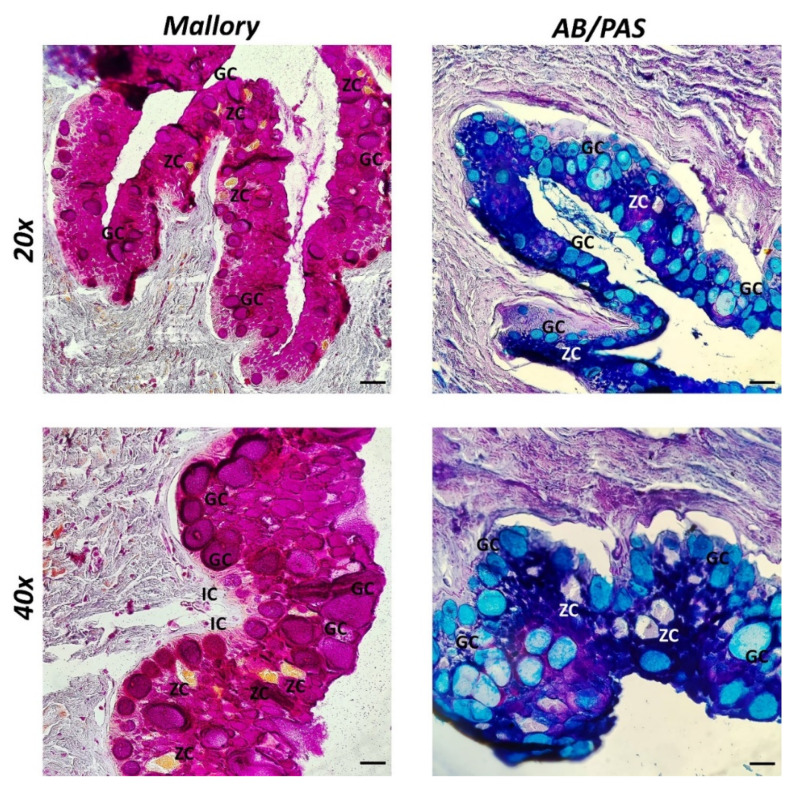
Intestine sections of broadgilled hagfish stained with Mallory Trichrome and AB/PAS, 20× and 40× scale bars of 20 and 40 µm. Zigzag ridges constitute the mucosa, which also contains scattered immune cells (IC) and visible zymogenous cells (ZC). Furthermore, goblet cells (GC) can be identified based on the type of secretion. Numerous ovoid-shaped goblet cells with acidophilic secretion are highlighted in blue by AB/PAS.

Three layers, the mucosa, submucosa, and muscularis serosa, are discernible in the intestine of broadgilled hagfish. A mucosa arranged in persistent, broad zigzag ridges was visible by Mallory histological staining. Zimogenous cells (ZC) were noted and are helpful for digestion. Immune cells are also scattered throughout the mucosa (IC) (Figure 1). Abundant goblet cells with an ovoid shape and acidophilic secretion were highlighted in blue using the histochemical staining method AB/PAS, which was used to differentiate goblet cells according to the type of secretion (acid in blue, neutral in magenta, and mixed in purple) (Figure 1).

**Figure 2:**
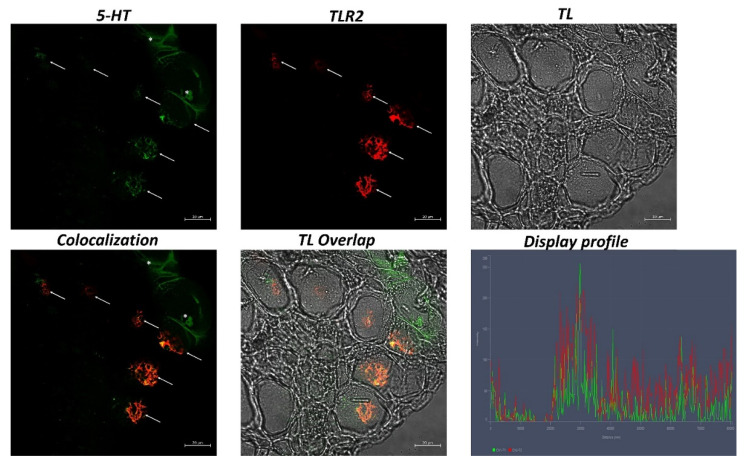
Immunofluorescence of 5-HT and TLR2 in the intestine of hagfish, 40×, scale bar 20 nm. Immunopositivity of goblet cells to 5-HT (green) and TLR2 (red) is evident (arrows). In addition, some neuroendocrine cells are highlighted with 5-HT (*). The colocalization of the two antibodies is confirmed by the “display profile” function. TL = transmitted light.

**Figure 3:**
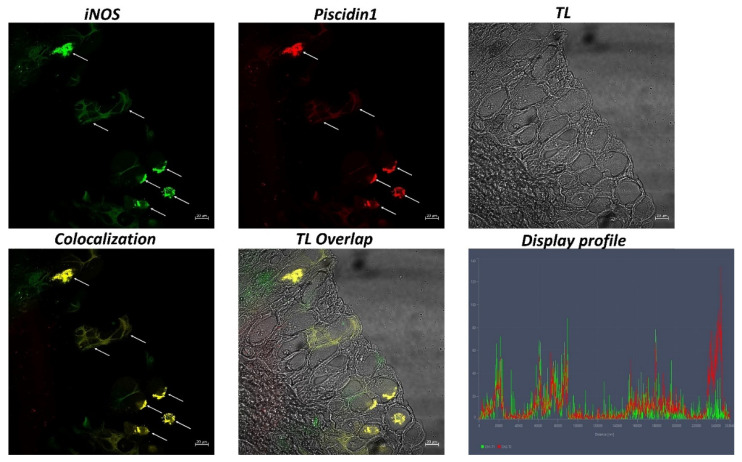
Immunofluorescence of iNOS and Piscidin1 in the intestine of hagfish, 40×, scale bar 20 nm. Immunopositivity of goblet cells to iNOS (green) and Piscidin1 (red) is noticeable (arrows). The colocalization of the two antibodies is confirmed by the “display profile” function. TL = transmitted light.

In the intestine sections of broadgilled hagfish, goblet cells positive to 5-HT and TLR2 were evident in the mucosa (Figure 2). Similarly, iNOS and Piscidin1 highlighted immunolabeled goblet cells in the intestinal epithelium (Figure 3). Strong colocalization between 5-HT and TLR2 and between iNOS and Piscidin1 can be seen, which is also supported by confocal microscopy graphs produced using the “display profile” function (Figure 2 and Figure 3).

Quantitative analysis revealed an equivalence of the number of goblet cells detected with the various antibodies (Table 2).

## 4. Discussion

The intestinal mucosa is crucial for the effective functioning of the digestive system and for maintaining its optimal absorption capacity, playing a key role in preserving fish body health [46,47]. As the literature confirms, goblet cells are involved in maintaining the functional and structural integrity of the intestinal epithelium [48,49,50]. The number of goblet cells in the intestinal epithelium might vary depending on the species, intestinal segment, dietary regimen, and stage of development [51].

The intestine of the myxines probably evolved from ancestral craniates with microphage feeding habits. As they lack distinct stomach or small- and large-intestine distinctions, lipases are used to carry out digestion directly inside the rectilinear intestinal tract. Three layers are present in the gut of myxines: mucosa, submucosa, and muscularis serosa [52].

According to our previous study, morphologically, there is a consistent mucosa, arranged in crests, with a lot of goblet cells, a submucosa, and a muscularis serosa [22]. AB/PAS identified various mucopolysaccharides, particularly in blue acidic mucins, as previously described in recent articles [14].

The neuroendocrine system is involved in maintaining intestinal health, stimulating the motility and the contraction of the intestine, and exciting the production of mucus by the goblet cells. In particular, a study by Engevik et al. (2019) suggested that serotonin, produced by neuroendocrine cells, can activate receptors 4 (5-HTR4) located on goblet cells to promote the secretion and release of mucins [17]. Goblet cells have been characterized by 5-HT in human [53] and fish [54]. A study by Lauriano et al. (2021) showed immunopositivity to 5-HT and Piscidin1 in gill and respiratory air-sack mucous cells in *Heteropneustes fossilis* [55]. The presence of 5-HT has already been demonstrated in hagfish in the nervous system [56], in chromaffin tissues [57], and in subcutaneous neurons [25]. Our results show goblet cells in the broadgilled hagfish intestine positive for 5-HT, suggesting phylogenetic preservation of this neurotransmitter in the stimulation of mucus secretion.

Goblet cells, as mentioned above, can also play an immune role [58,59]. Recent research shows that some goblet cells present TLR receptors on their surface and in the cytoplasm, playing a sentinel role and stimulating mucus production [10,60]. In some studies conducted on *Danio rerio*, it was noted that TLR2, solicited by microbial stimuli, can activate goblet cells for the production and secretion of protective mucus [61,62]. We have recently immunohistochemically demonstrated the presence of TLR2 in the intestine of broadgilled hagfish [22]. Our results show goblet cells in the gut of the broadgilled hagfish immunopositive to TLR2, suggesting that some goblet cells may also play a sentinel function in this ancestral vertebrate.

iNOS plays a role in the regulation and secretion of mucus by goblet cells. Williams et al. (2008) demonstrated the phylogenetic presence of all isoforms of NOS in fish classes from cyclostomes to teleosts [63]. A study by Torrecillas et al. (2017) on *Dicentrarchus labrax* hypothesized that iNOS-positive goblet cells may also be involved in the production of these important neuroendocrine and immune molecules [64]. Pederzoli et al. (2007) showed the presence of two NOS isoforms (nNOS and iNOS) in the intestine of *Dicentrarchus labrax*, to iNOS immune and enteroendocrine roles in intestinal stimulation to iNOS [65].

In fish, mucus secreted by goblet cells is rich in mucins, esterases, proteases, complement proteins, and antimicrobial peptides [5,66]. AMPs are molecules produced by almost all organisms. In fish, they include piscidins, defensins, hepcidins, and cathelicidins. Piscidins are among the most powerful and broad-spectrum AMPs and are highly conserved between fish, commonly occurring in the gills, muscle, cephalic kidney, skin, and intestine [67]. They have been found in mast cells, rodlet cells, mucous epithelial cells, phagocytes, and intestinal goblet cells [68]. The antibacterial activity of fish mucus was confirmed by Tiralongo et al. (2020) in a study on the mucus of haddock (*Melanogrammus aeglefinus*), striped bass (*Morone sacsatilis*), arctic char (*Salvelinus alpinus*), koi carp (*Cyprinus carpio*), brook trout (*Salvelinus fontinalis*), and hagfish (*Myxine glutinosa*) [69]. We have characterized goblet cells in the broadgilled hagfish intestine with Piscidin1, according to previous studies [55,68].

In conclusion, our results characterize immunohistochemically intestinal goblet cells of broadgilled hagfish for the first time with the following antibodies, 5-HT/TLR2 and iNOS/Piscidin1, suggesting the hypothesis of conservation of the roles played by these cells also in primitive vertebrates. Marked colocalization between 5-HT and TLR2 and between iNOS and Piscidin1 may indicate a phylogenetic correlation in the involvement of these molecules in goblet cell function. In this study, monoclonal (5-HT and iNOS) and polyclonal (TLR2 and Piscidin1) antibodies were used. It is known that polyclonal antibodies may be less specific than monoclonal antibodies. This may induce cross-reactivity and possible false positivity. Although the data we collected are effectively supported by the scientific literature and previous studies, future in-depth studies may be useful to further corroborate our analyses. Furthermore, these results may contribute to a deeper understanding of these particular and fundamental intestinal cells and to expanding knowledge about the immune system of broadgilled hagfish, a “vertebrate” about which still little is known. However, this research may contribute as a base for further studies, which are necessary in order to confirm the involvement of goblet cells in the intestinal immunity of hagfish.

## Figures and Tables

**Table 1:** Antibodies data.

Antibody	Supplier	Dilution	Animal Source
5-HT	Santa Cruz Biotechnology, Inc., Dallas, TX, USA	1:50	Mouse
TLR2	Active Motif, La Hulpe, Belgium, Europe	1:125	Rabbit
iNOS	Santa Cruz Biotechnology, Inc., Dallas, TX, USA	1:200	Mouse
Piscidin1	GenScript Biotech Corporation, Rijswijk, Netherlands, Europe. Produced on demand	1:50	Rabbit
Alexa Fluor 488 donkey anti-mouse IgG FITC conjugated	Molecular Probes, Invitrogen	1:300	Donkey
Alexa Fluor 594 donkey anti-rabbit IgG TRITC conjugated	Molecular Probes, Invitrogen	1:300	Donkey

**Table 2:** Quantitative analysis data (mean values ± standard deviation; *n* = 3).

	No. of Goblet Cells ^1^
5-HT+	356.71 ± 24.54 *
TLR2+	294.29 ± 19.02 *
5-HT + TLR2	263.43 ± 28.68 *
iNOS+	325.02 ± 24.94 **
Piscidin1+	385.38 ± 28.05 *
iNOS+Piscidin1	308.39 ± 15.14 *

** p ≤ 0.01, * p ≤ 0.05; ^1^ One-way ANOVA and Student’s *t* test were used to compare the means.

## Data Availability

The data presented in this study are available within the article.
